# Prevalence and prognostic impact of unrecognized myocardial infarction detected by cardiac magnetic resonance in Thai patients with obesity

**DOI:** 10.1371/journal.pone.0353109

**Published:** 2026-07-06

**Authors:** Punyanuch Chayanopparat, Piyachai Siriphiphatcharoen, Issarayus Laohabut, Thanawat Ruengchaisiwawaith, Yodying Kaolawanich

**Affiliations:** Division of Cardiology, Department of Medicine, Faculty of Medicine Siriraj Hospital, Mahidol University, Bangkok, Thailand; Sichuan University, CHINA

## Abstract

**Background:**

Unrecognized myocardial infarction (UMI) is associated with adverse outcomes and may occur in patients with obesity, who are at increased cardiovascular risk. Although cardiac magnetic resonance (CMR) enables accurate detection of UMI, prognostic data in patients with obesity remain limited, particularly in Asian populations. This study evaluated the prevalence and prognostic impact of CMR-detected UMI in Thai patients with obesity.

**Methods:**

This cohort study included 1,053 patients with BMI ≥ 25 kg/m^2^, without a prior diagnosis of myocardial infarction (MI) or coronary revascularization (mean age, 67 ± 11 years; 71.3% grade 1 and 28.7% grade 2 obesity), who underwent CMR at an academic hospital in Thailand between 2014 and 2016. The most common indication was suspected coronary artery disease (78.3%). UMI was identified using late gadolinium enhancement imaging. Patients were followed for major adverse cardiovascular events (MACE), defined as a composite of cardiovascular death, nonfatal MI, or hospitalization for heart failure.

**Results:**

UMI was identified in 105 patients (9.9%). During a median follow-up of 9.2 (5.2–10.2) years, patients with UMI had a significantly higher rate of MACE compared with those without UMI (2.58 vs. 0.89 per 100 patient-years; HR, 2.86; 95% CI, 1.68–4.88; p < 0.001). In multivariable analysis, age, diabetes, cigarette smoking, history of heart failure, LVEF, and UMI were independently associated with MACE. The presence of UMI provided incremental prognostic value beyond traditional risk factors (Δχ^2^ = 22.43; p < 0.001). Across all subgroups defined by age, sex, obesity grade, diabetes status, symptoms, LVEF, and myocardial ischemia, UMI was consistently associated with higher risks of MACE, with no significant interactions observed (all p for interaction>0.05).

**Conclusions:**

In Thai patients with obesity undergoing CMR, the prevalence of UMI was 9.9% and was independently associated with MACE, providing incremental prognostic value. These findings suggest that CMR-detected UMI may play a role in risk stratification in this population.

## Introduction

Unrecognized myocardial infarction (UMI) refers to myocardial infarction (MI) that occurs without clinical recognition at the time of onset. Although asymptomatic, UMI carries prognostic implications similar to recognized MI, including higher risks of heart failure, recurrent ischemic events, and cardiovascular death [1–4].

Obesity is a major cardiovascular risk factor associated with cardiac structural remodeling, myocardial dysfunction, and adverse metabolic profiles. Late gadolinium enhancement (LGE) cardiac magnetic resonance (CMR) is the reference standard for detecting myocardial scar and UMI, whereas conventional tools such as electrocardiography (ECG), echocardiography, and single-photon emission computed tomography (SPECT) have limited sensitivity [1–6]. Although UMI can occur across the body mass index (BMI) spectrum, its detection in obese individuals may be particularly challenging due to atypical or masked symptoms and body habitus.

The relationship between obesity and UMI remains controversial. A multicenter EuroCMR study by Jensen et al. demonstrated that while obese patients had a slightly higher unadjusted prevalence of unrecognized scar and MI compared to normal-weight individuals (13.7% versus 9.1% and 10.3% versus 7.4%, respectively), these differences were not significant after adjustment for Framingham risk factors, suggesting a possible “obesity paradox” [7]. Nevertheless, the prognostic impact of CMR-detected UMI in obese patients remains unclear, especially in the Asian population, such as Thais, whose obesity thresholds and risk profiles differ [8,9].

This study aimed to determine the prevalence and prognostic impact of CMR-detected UMI in Thai patients with obesity for predicting major adverse cardiovascular events (MACE), defined as a composite of cardiovascular death, nonfatal MI, and hospitalization for heart failure.

## Methods

### Study population

This study included consecutive patients aged 18 years or older with obesity who were referred for CMR at the Division of Cardiology, Department of Medicine, Faculty of Medicine Siriraj Hospital, Mahidol University, Bangkok, Thailand, between 2014 and 2016. The definition and classification of obesity were determined according to the World Health Organization, the International Association for the Study of Obesity, and the International Obesity Task Force Asia-Pacific Perspective [8]. Obesity grade 1 was defined as a BMI of 25.0–29.9 kg/m^2^, and grade 2 was defined as a BMI of ≥30.0 kg/m^2^ [8]. Exclusion criteria included a history of MI, prior coronary revascularization, severe valvular heart disease, specific non-ischemic cardiomyopathies (e.g., hypertrophic or infiltrative cardiomyopathy), congenital heart disease, incomplete or missing CMR data, and lack of follow-up data. Additionally, approximately 10% of patients with a normal BMI (<25 kg/m^2^) who were randomly selected during the same study period were also included for comparison (data were presented in the supporting information files).

The study protocol conformed to the ethical guidelines of the 1975 Declaration of Helsinki. This study was approved by the Siriraj Institutional Review Board, Faculty of Medicine Siriraj Hospital, Mahidol University (COA no. Si 746/2025). The requirement for informed consent was waived by the board because of the retrospective study design and the removal of all personal identifying information. Data were accessed for research purposes between October 1, 2025, and November 30, 2025.

### Patients follow‑up and clinical outcome

Follow-up data were collected from medical records (last follow-up: September 7, 2025). The primary outcome was the occurrence of MACE, defined as a composite of cardiovascular death, nonfatal MI, and hospitalization for heart failure. Cardiovascular death was defined in accordance with established published criteria [10]. In cases of multiple events, only the initial event was counted for event-free survival analysis.

### CMR protocol and image analysis [11–13]

CMR studies were performed using a 1.5 or 3.0 Tesla Philips Achieva XR scanner (Philips Medical Systems, Best, The Netherlands) to evaluate cardiac function, LGE, and vasodilatory stress perfusion. Images for the cardiac functional study were acquired using the steady-state free precession (SSFP) technique in multiple short-axis slices, as well as in 2-chamber, 3-chamber, and 4-chamber views. The imaging parameters for the cardiac functional study at 1.5 Tesla were as follows: echo time (TE) of 1.8 milliseconds (ms), repetition time (TR) of 3.7 ms, number of excitations of 2, field of view (FOV) of 390 × 312 mm, matrix size of 256 × 240, reconstruction pixel size of 1.52 × 1.21 mm, slice thickness of 8 mm, and a flip angle of 70 degrees. LGE images were acquired 10 minutes after administration of gadolinium (49% gadoterate meglumine, 46% gadopentetate dimeglumine, 5% other agents) at a dose of 0.1 mmol/kg and a rate of 4 mL/s, using a 3D segmented gradient-echo inversion-recovery sequence. These images were obtained in multiple short-axis slices, as well as in long-axis, 2-chamber, and 4-chamber views, similar to the functional images. Image parameters at 1.5 Tesla included a TE of 1.25 ms, TR of 4.1 ms, a flip angle of 15 degrees, a FOV of 303 × 384 mm, matrix size of 240 × 256, in-plane resolution of 1.26 × 1.5 mm, slice thickness of 8 mm, and a sensitivity encoding factor of 1.5. Vasodilatory stress perfusion tests were performed before LGE imaging, and the CMR protocol followed standard procedures described elsewhere [11].

Quantitative measurements of left ventricular (LV) volume, and ejection fraction (EF) were obtained from the stack of short-axis SSFP cine images. MI on LGE images was identified by visual assessment of hyperenhanced areas. LGE was considered present only when confirmed in both the short-axis view and at least one other orthogonal plane [13]. LGE patterns were categorized as MI when subendocardial or transmural. The total number of LGE segments was determined utilizing the American Heart Association 17-segment model [14]. Stress perfusion images were examined with visualization of each of the 16 myocardial segments (excluding apical segment 17). Inducible myocardial ischemia was defined as a subendocardial perfusion defect meeting the following criteria: persistence beyond peak myocardial enhancement for several RR intervals, a width exceeding two pixels, alignment with one or more coronary artery territories, and absence of LGE in the corresponding segment [13].

### Statistical analysis

Continuous variables are presented as mean±standard deviation or median (interquartile range [IQR]), as appropriate, and categorical variables as frequencies and percentages. Comparisons between groups were performed using the Student’s t-test or Mann–Whitney U test for continuous variables and the chi-square or Fisher’s exact test for categorical variables, as appropriate. Univariable and multivariable logistic regression analyses were used to identify clinical predictors of UMI, with results reported as odds ratios (ORs) and 95% confidence intervals (CIs). Time-to-event analyses for MACE were performed using Kaplan–Meier survival curves and compared with the log-rank test. Cox proportional hazards regression models were constructed to identify independent predictors of MACE, with results expressed as hazard ratios (HRs) and 95% CIs. Two multivariable Cox models were developed: Model 1 included significant variables from univariable analysis (p < 0.05) using backward selection, and Model 2 included traditional coronary risk factors with forced entry of UMI. Incremental prognostic value was assessed by changes in global chi-square (Δχ^2^). Receiver operating characteristic (ROC) curve analysis was performed to evaluate the predictive performance of the number of UMI segments for predicting MACE. The area under the curve (AUC) was calculated, and sensitivity and specificity were derived across cutoff values. The optimal cutoff was determined using the Youden index. Subgroup analyses were conducted to assess effect consistency across subgroups, with interaction testing. A two-sided p value <0.05 was considered statistically significant. All statistical analyses were performed using SPSS software, version 20.0 (IBM Corp., Armonk, NY, USA).

## Results

### Patient characteristics

**Fig 1:**
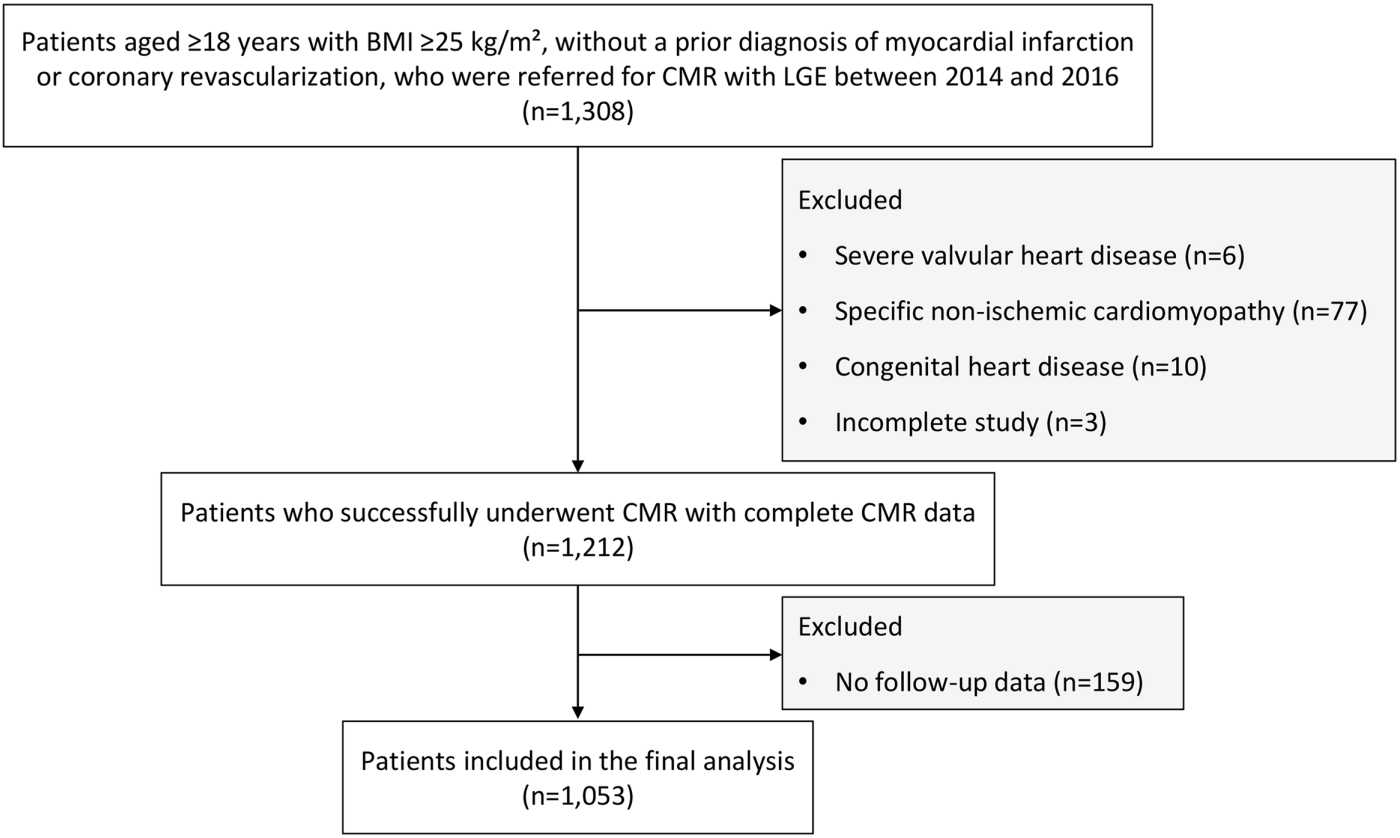
Study flow chart. Flow diagram illustrating patient selection and exclusion criteria. Among patients referred for clinical CMR, those meeting the inclusion criteria were enrolled in the final study cohort of patients with obesity and followed for clinical outcomes. Abbreviations: CMR, cardiac magnetic resonance.

Fig 1 shows the study flow chart. Among 1,308 patients aged ≥18 years with BMI ≥ 25 kg/m^2^ who were referred for clinical CMR, 6 were excluded due to severe valvular heart disease, 77 due to specific non-ischemic cardiomyopathies, 10 due to congenital heart disease, 3 due to incomplete CMR studies, and 159 due to lack of follow-up data. A total of 1,053 patients with obesity were included in the final analysis. Reasons for CMR included suspected coronary artery disease (CAD) (78.3%); abnormal prior cardiac testing (9.7%); preoperative evaluation for non-cardiac surgery (6.3%); and other indications (5.7%).

Table 1 summarizes the baseline characteristics of the study population. The mean age was 67.1 ± 11.3 years, and 41.3% were male. The mean BMI was 28.9 ± 3.3 kg/m^2^, comprising 71.3% grade 1 and 28.7% grade 2 obesity. A total of 422 patients (40.1%) had diabetes mellitus, 94 (8.9%) had a history of heart failure, and dyspnea was the most common presenting symptom (59.2%). The mean LVEF was 68.8 ± 12.7%.

**Table 1:** Baseline characteristics of patients with obesity with and without UMI.

	Total	UMI	No UMI	P-value
	(n = 1,053)	(n = 105)	(n = 948)	
Age, years	67.1 ± 11.3	66.3 ± 13.4	67.3 ± 11.0	0.49
Male	435 (41.3)	73 (69.5)	362 (38.2)	***<0.001***
BMI, kg/m^2^	28.9 ± 3.3	28.7 ± 3.1	29.0 ± 3.3	0.42
Obesity grade				0.24
1 (BMI 25–29.9 kg/m^2^)	751 (71.3)	80 (76.2)	671 (70.8)	
2 (BMI ≥ 30 kg/m^2^)	302 (28.7)	25 (23.8)	277 (29.2)	
Medical history				
Hypertension	882 (83.8)	88 (83.8)	794 (83.8)	0.98
Diabetes mellitus	422 (40.1)	50 (47.6)	372 (39.2)	0.09
Hyperlipidemia	911 (86.5)	99 (94.3)	812 (85.7)	***0.01***
Family history of CAD	24 (2.3)	0 (0.0)	24 (2.5)	0.16
Cigarette smoking	102 (9.7)	24 (22.9)	78 (8.2)	***<0.001***
History of heart failure	94 (8.9)	20 (19.0)	74 (7.8)	***<0.001***
Atrial fibrillation	129 (12.3)	11 (10.5)	118 (12.4)	0.56
Ischemic stroke	75 (7.1)	10 (9.5)	65 (6.9)	0.31
Symptoms				
Chest pain	380 (36.1)	37 (35.2)	343 (36.2)	0.84
Dyspnea	623 (59.2)	58 (55.2)	565 (59.7)	0.38
Others	66 (6.3)	5 (4.8)	61 (6.4)	0.50
12-lead ECG ^a^				
Q waves	100 (10.9)	25 (26.0)	75 (9.1)	***<0.001***
Medications				
Aspirin	478 (45.4)	69 (65.7)	409 (43.1)	***<0.001***
ACE inhibitor or ARB	511 (48.5)	64 (61.0)	447 (47.2)	***0.007***
Beta blocker	526 (50.0)	62 (59.0)	464 (48.9)	***0.04***
Calcium channel blocker	388 (36.8)	37 (35.2)	351 (37.0)	0.72
Statin	630 (59.8)	79 (75.2)	551 (58.1)	***0.001***
Oral antidiabetic drug	261 (24.8)	27 (25.7)	234 (24.7)	0.81
Insulin	35 (3.3)	7 (6.7)	28 (3.0)	0.07
CMR				
LVEDV index, mL/m^2^	73.5 ± 24.2	100.2 ± 44.3	70.5 ± 18.6	***<0.001***
LVESV index, mL/m^2^	25.2 ± 22.3	52.4 ± 45.3	22.13 ± 15.3	***<0.001***
LVEF, %	68.8 ± 12.7	54.4 ± 19.4	70.3 ± 10.6	***<0.001***
Presence of myocardial ischemia	154 (14.6)	70 (66.7)	84 (8.9)	***<0.001***
Number of segments of myocardial ischemia ^b^	5 (3,7)	5 (3,8)	5 (3,7)	0.81
Number of segments of UMI ^c^	3 (2,6)	3 (2,6)	0 (0,0)	***<0.001***

Data are presented as mean±standard deviation, median (interquartile range), or number (percentage), as appropriate. Bold italic values indicate statistical significance (p < 0.05).

^a^ ECG data were available in 917 patients.

^b^ Data available in patients with myocardial ischemia.

^c^Data available in patients with UMI.

Abbreviations: ACE, angiotensin-converting enzyme; ARB, angiotensin receptor blocker; BMI, body mass index; CAD, coronary artery disease; CMR, cardiac magnetic resonance; ECG, electrocardiography; LVEDV, left ventricular end-diastolic volume; LVESV, left ventricular end-systolic volume; LVEF, left ventricular ejection fraction; UMI, unrecognized myocardial infarction.

UMI was present in 105 (9.9%) patients, with a median number of UMI segments of 3 (IQR 2–6). Patients with UMI were more often male and had a higher prevalence of hyperlipidemia, cigarette smoking, a history of heart failure, and more frequent Q waves on ECG compared with those without UMI (p < 0.05 for all). For CMR parameters, patients with UMI had significantly lower LVEF (54.4 ± 19.4 versus 70.3 ± 10.6, p < 0.001) and a higher prevalence of myocardial ischemia on stress perfusion imaging (66.7% versus 8.9%, p < 0.001).

In patients with normal BMI (n = 191; mean BMI, 22.3 ± 2.1 kg/m^2^), UMI was identified in 31 patients (16.2%). S1 Table summarizes the baseline characteristics of patients with normal BMI with and without UMI.

### Predictors of UMI

Table 2 demonstrates the univariable and multivariable logistic regression analyses for the clinical prediction of UMI. In univariable analysis, male sex, hyperlipidemia, cigarette smoking, history of heart failure, and Q waves on ECG were associated with UMI (all p < 0.05). In multivariable analysis, male sex (OR, 3.40; 95% CI, 2.14–5.40; p < 0.001), hyperlipidemia (OR, 2.79; 95% CI, 1.17–6.65; p = 0.02), history of heart failure (OR, 3.00; 95% CI, 1.65–5.44; p < 0.001), and Q waves (OR, 3.43; 95% CI, 1.99–5.90; p < 0.001) were independently associated with UMI.

**Table 2:** Univariable and multivariable logistic regression analyses for predictors of UMI.

	Univariable analysis			Multivariable analysis		
Variables	OR	95%CI	P-value	OR	95%CI	P-value
Age	0.99	0.97, 1.01	0.42			
Male	3.69	2.39, 5.71	***<0.001***	3.40	2.14, 5.40	***<0.001***
BMI	0.97	0.91, 1.04	0.64			
Obesity grade	0.76	0.47, 1.21	0.24			
Hypertension	1.004	0.58, 1.73	0.98			
Diabetes mellitus	1.40	0.94, 2.11	0.09			
Hyperlipidemia	2.76	1.19, 6.42	***0.01***	2.79	1.17, 6.65	***0.02***
Family history of CAD	N/A	N/A	N/A			
Cigarette smoking	3.30	1.98, 5.51	***<0.001***			
History of heart failure	2.78	1.61, 4.78	***<0.001***	3.00	1.65, 5.44	***<0.001***
Atrial fibrillation	0.82	0.42, 1.58	0.34			
Ischemic stroke	1.43	0.71, 2.87	0.31			
Chest pain	0.96	0.63, 1.46	0.84			
Dyspnea	0.83	0.55, 1.25	0.38			
Q waves on ECG	3.51	2.10, 5.88	***<0.001***	3.43	1.99, 5.90	***<0.001***

Bold italic values indicate statistical significance (p < 0.05).

Abbreviations: CAD, coronary artery disease; CI, confidence interval; ECG, electrocardiography; N/A, not available; OR, odds ratio; UMI, unrecognized myocardial infarction.

### Patient outcomes

Among patients with UMI, 60 underwent coronary angiography, and 50 subsequently underwent coronary revascularization. In addition, 57 patients had medication adjustments, primarily involving the addition or up-titration of therapy, including antiplatelet agents (n = 14), antihypertensive medications (n = 37), glucose-lowering therapy (n = 14), and statins (n = 16).

**Fig 2:**
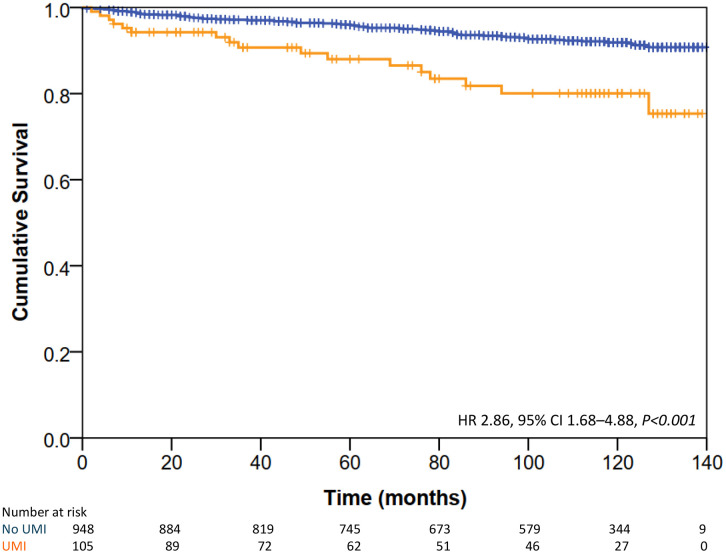
Kaplan–Meier curves for MACE. Kaplan–Meier curves showing the survival free of MACE, stratified by the presence or absence of UMI detected by CMR. Patients with UMI had a significantly higher incidence of MACE than those without UMI (log-rank P < 0.001). Abbreviations: CMR, cardiac magnetic resonance; MACE, major adverse cardiovascular events; UMI, unrecognized myocardial infarction.

During a median follow-up of 9.2 (5.2–10.2) years, 83 MACE occurred (5 cardiovascular deaths, 34 nonfatal MIs, and 59 hospitalizations for heart failure). Table 3 demonstrates details of patient outcomes and compares patients with and without UMI. Patients with UMI had a significantly higher rate of MACE compared with those without UMI (2.58 versus 0.89 per 100 patient-years; HR, 2.86; 95% CI, 1.68–4.88; p < 0.001). Kaplan–Meier analysis for MACE in patients with obesity, stratified by the presence or absence of UMI is shown in Fig 2 (log-rank p < 0.001). Among patients with normal BMI, those with UMI also had a significantly higher rate of MACE than those without UMI (log-rank p < 0.001) (S1 Fig).

**Table 3:** Patient outcomes.

	Total	UMI	No UMI	HR	95% CI	P-value
	(n = 1,053)	(n = 105)	(n = 948)			
MACE	83 (7.9)	17 (16.2)	66 (7.0)	2.86	1.68, 4.88	***<0.001***
Cardiovascular death	5 (0.5)	3 (2.9)	2 (0.2)	15.99	2.66, 95.01	***0.002***
Nonfatal MI	34 (3.2)	6 (5.7)	28 (3.0)	2.36	0.98, 5.71	0.05
Hospitalization for heart failure	59 (5.6)	11 (10.5)	48 (5.1)	2.51	1.30, 4.82	***0.006***

Bold italic values indicate statistical significance (p < 0.05).

Abbreviations: CI, confidence interval; HR, hazard ratio; MACE, major adverse cardiovascular events; MI, myocardial infarction; UMI, unrecognized myocardial infarction.

### Cox regression analyses and incremental prognostic value testing of UMI for MACE

Table 4 demonstrates the univariable Cox regression analysis of clinical and CMR characteristics for the prediction of MACE in patients with obesity. Age, diabetes mellitus, cigarette smoking, history of heart failure, atrial fibrillation, early coronary revascularization (<90 days after CMR), left ventricular end-diastolic volume index (LVEDVI), left ventricular end-systolic volume index (LVESVI), LVEF, presence and extent of myocardial ischemia, and number of UMI segments were significantly associated with MACE (all p < 0.05).

**Table 4:** Univariable Cox regression analysis of clinical and CMR characteristics for the prediction of MACE.

Variables	HR	95%CI	P-value
Age, years	1.03	1.01, 1.05	***0.002***
Male	1.23	0.80, 1.90	0.34
BMI, kg/m^2^	1.04	0.98, 1.10	0.17
Obesity grade	1.38	0.88, 2.17	0.16
Hypertension	1.83	0.88, 3.80	0.10
Diabetes mellitus	1.66	1.08, 2.56	***0.02***
Hyperlipidemia	0.93	0.49, 1.76	0.83
Family history of CAD	0.45	0.06, 3.24	0.42
Cigarette smoking	2.15	1.23, 3.77	***0.007***
History of heart failure	3.81	2.26, 6.43	***<0.001***
Atrial fibrillation	1.77	1.03, 3.06	***0.04***
Ischemic stroke	1.54	0.74, 3.20	0.24
Chest pain	0.92	0.58, 1.45	0.72
Dyspnea	1.37	0.87, 2.16	0.17
Q waves on 12-lead ECG	1.52	0.80, 2.89	0.20
Early revascularization within 90 days of CMR	2.04	1.02, 4.08	***0.04***
LVEDV index, mL/m^2^	1.01	1.01, 1.02	***<0.001***
LVESV index, mL/m^2^	1.01	1.01, 1.02	***<0.001***
LVEF, %	0.97	0.95, 0.98	***<0.001***
Presence of myocardial ischemia	2.74	1.70, 4.40	***<0.001***
Number of segments of myocardial ischemia	1.11	1.05, 1.18	***<0.001***
Number of segments of UMI	1.26	1.17, 1.36	***<0.001***

Bold italic values indicate statistical significance (p < 0.05).

Abbreviations: BMI, body mass index; CAD, coronary artery disease; CI, confidence interval; CMR, cardiac magnetic resonance; ECG, electrocardiography; HR, hazard ratio; LVEDV, left ventricular end-diastolic volume; LVESV, left ventricular end-systolic volume; LVEF, left ventricular ejection fraction; MACE, major adverse cardiovascular events; UMI, unrecognized myocardial infarction.

Table 5 shows multivariable Cox regression models for the prediction of MACE and incremental value testing. Two multivariable models were constructed. Model 1 included significant predictors from the univariable analysis (Table 4) using the backward selection method. Model 2 included traditional coronary risk factors (age, sex, hypertension, diabetes mellitus, hyperlipidemia, and cigarette smoking) and UMI using the enter method. The number of UMI segments was an independent predictor of MACE in both multivariable models (Model 1: HR, 1.11; 95% CI, 1.01–1.22; p = 0.03; Model 2: HR, 1.26; 95% CI, 1.17–1.37; p < 0.001).

**Table 5:** Multivariable Cox regression models for the prediction of MACE and incremental value testing.

	Variables	Model 1 ^a^			Variables	Model 2 ^b^		
		HR	95%CI	P-value		HR	95%CI	P-value
Multivariable	Age, years	1.04	1.02, 1.07	***<0.001***	Age, years	1.04	1.01, 1.06	***0.001***
	Diabetes mellitus	1.60	1.04, 2.48	***0.03***	Male	0.89	0.54, 1.46	0.64
	Cigarette smoking	1.87	1.03, 3.40	***0.04***	Hypertension	1.35	0.63, 2.89	0.42
	History of heart failure	2.55	1.45, 4.49	***0.001***	Diabetes mellitus	1.62	1.04, 2.53	***0.03***
	LVEF, %	0.98	0.96, 0.99	***0.02***	Hyperlipidemia	0.65	0.33, 1.25	0.19
	Number of segments of UMI	1.11	1.01, 1.22	***0.03***	Cigarette smoking	2.14	1.12, 4.09	***0.02***
					Number of segments of UMI	1.26	1.17, 1.37	***<0.001***
Incremental value testing	Model 1 without UMI	χ^2^ 66.70, ***P < 0.001***			Model 2 without UMI	χ^2^ 24.87, ***P < 0.001***		
	Model 1 with UMI	χ^2^ 88.76, ***P < 0.001***			Model 2 with UMI	χ^2^ 68.35, ***P < 0.001***		
	Incremental value	χ^2^ 6.32, ***P=0.01***			Incremental value	χ^2^ 22.43, ***P < 0.001***		

^a^Model 1 included significant variables from univariable analysis (p < 0.05) using backward selection.

^b^Model 2 included traditional coronary risk factors with forced entry of UMI.

Bold italic values indicate statistical significance (p < 0.05).

Abbreviations: CI, confidence interval; HR, hazard ratio; LVEF, left ventricular ejection fraction; MACE, major adverse cardiovascular events; UMI, unrecognized myocardial infarction.

**Fig 3:**
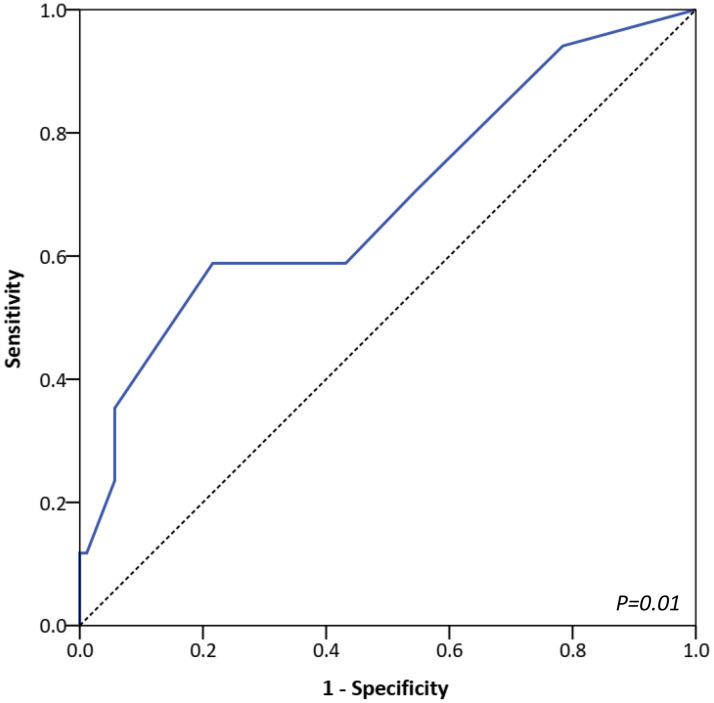
ROC analysis of UMI burden for predicting MACE. ROC curve demonstrates the predictive performance of the number of UMI segments for MACE. The AUC was 0.70 (95% CI, 0.54–0.84; p = 0.01). A cutoff value of ≥6 UMI segments, determined by the Youden index, yielded a sensitivity of 0.60 and a specificity of 0.78 for predicting MACE. Abbreviations: AUC, area under the curve; MACE, major adverse cardiovascular events; ROC, receiver operating characteristic; UMI, unrecognized myocardial infarction.

The addition of UMI to the models resulted in a significant increase in global χ^2^ for both Model 1 and Model 2 (Δχ^2^ = 6.32; p = 0.01 and Δχ^2^ = 22.43; p < 0.001, respectively). Fig 3 shows the ROC analysis of the number of UMI segments for predicting MACE, demonstrating an AUC of 0.70 (95% CI, 0.54–0.84; p = 0.01). A cutoff value of ≥6 segments, determined by the Youden index, yielded a sensitivity of 0.61 and a specificity of 0.78 for predicting MACE.

### Subgroup analysis

**Fig 4:**
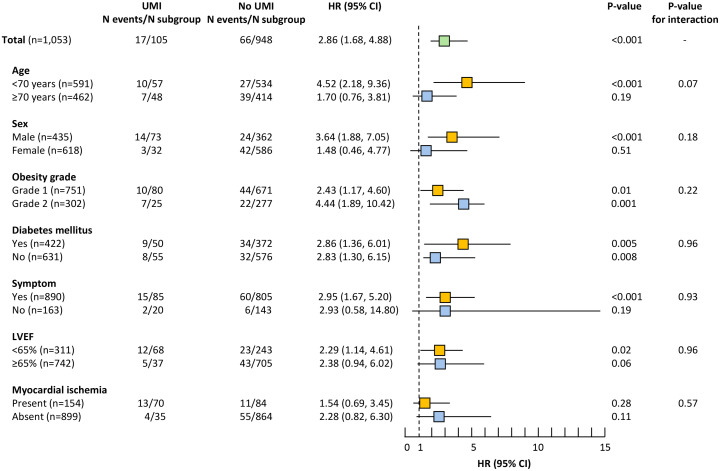
Subgroup analysis of the association between UMI and MACE. Forest plot showing HRs for MACE associated with UMI across predefined subgroups, including age, sex, obesity grade, diabetes status, symptoms, LVEF, and myocardial ischemia. The association between UMI and MACE was consistent across all subgroups, with no significant interactions observed (all p for interaction >0.05). Abbreviations: HRs, hazard ratios; LVEF, left ventricular ejection fraction; MACE, major adverse cardiovascular events; UMI, unrecognized myocardial infarction.

Fig 4 illustrates seven subgroup analyses for MACE. Subgroup analyses based on age, sex, obesity grade, diabetes status, symptoms, LVEF, and myocardial ischemia consistently demonstrated higher HRs for MACE among patients with UMI compared with those without UMI, with no significant interactions observed across all subgroups (all p-values for interaction >0.05).

## Discussion

In this retrospective cohort of Thai patients with obesity undergoing CMR, the prevalence of CMR-detected UMI was 9.9% and was independently associated with MACE, defined as a composite of cardiovascular death, nonfatal MI, and hospitalization for heart failure. Importantly, the prognostic impact of UMI remained significant after adjustment for established clinical risk factors and other CMR parameters, and UMI provided incremental prognostic value beyond traditional cardiovascular risk models. These findings extend existing evidence on the clinical relevance of UMI and provide novel insights into its prognostic significance in patients with obesity.

The prevalence of UMI in our cohort was 9.9%, which is broadly consistent with prior CMR-based studies in mixed or Western populations [7]. However, direct comparisons across studies are limited by differences in population characteristics, obesity definitions, referral indications, and exclusion criteria. In addition, because all patients in the present study were referred for clinical CMR, referral bias may limit generalizability to the broader population with obesity. In Asian populations, obesity is defined using lower BMI thresholds than in Western cohorts, reflecting differences in body composition, visceral adiposity, and cardiometabolic risk profiles [8,9]. These differences may influence both the prevalence and clinical impact of UMI, underscoring the importance of population-specific data.

The prevalence of UMI in patients with obesity in our study was lower than that in patients with normal BMI (16.2%). Although this finding may be influenced by confounding factors, it may reflect the obesity paradox, whereby despite obesity being a major cardiovascular risk factor, obese patients may not have higher rates of UMI [7] and may experience better survival during acute coronary events compared with normal-weight individuals [15]. Nevertheless, the prognostic value of CMR-detected UMI was consistent in both patients with obesity and those with normal BMI.

The prognostic value of CMR-detected UMI has been well established. An early landmark study by Kwong et al. demonstrated that among 195 patients with signs or symptoms of CAD but without a history of MI who underwent CMR, 44 had LGE [16]. LGE was independently associated with MACE and cardiac mortality and provided incremental prognostic value [16]. A subsequent larger study by Antiochos et al., including 2,349 patients who underwent stress CMR, demonstrated that the presence of UMI or clinically recognized MI conferred a similarly increased risk of death and/or MI, independent of the presence of ischemia [17]. Together, these findings confirm the prognostic value of CMR-detected UMI.

Our study in a specific population of Thai patients with obesity demonstrates that CMR-detected UMI is a strong predictor of adverse cardiovascular outcomes. The presence of UMI was associated with an approximately threefold increased risk of MACE. This association remained significant after multivariable adjustment, indicating that UMI provides prognostic information beyond traditional clinical and other CMR parameters. These findings are consistent with prior CMR studies showing that unrecognized myocardial scar or UMI confers a risk of adverse outcomes comparable to that of clinically recognized MI [1–4]. The underlying mechanisms are likely multifactorial and include systemic inflammation, insulin resistance, microvascular dysfunction, and myocardial lipotoxicity. In patients with obesity, these processes may be further amplified [18].

Importantly, UMI was associated not only with the composite endpoint of MACE but also with individual outcomes, particularly cardiovascular death and hospitalization for heart failure. The strong association between UMI and heart failure hospitalization is clinically relevant, as obesity-related heart failure represents a growing public health burden in Asia [19,20]. The presence of UMI may predispose patients to progressive ventricular dysfunction and clinical decompensation, even in the absence of overt ischemic symptoms.

An additional key finding of our study is the incremental prognostic value of UMI beyond established risk factors. The addition of UMI to multivariable Cox regression models resulted in a significant increase in the global chi-square, indicating improved model performance. Furthermore, the number of UMI segments emerged as an independent predictor of MACE, indicating that a greater myocardial scar burden was associated with adverse outcomes. ROC analysis demonstrated moderate discriminative ability of UMI segment number for predicting MACE, with an optimal cutoff of six segments identified. Although this threshold should be interpreted cautiously, it suggests that myocardial scar burden may help refine risk stratification in selected patients.

Subgroup analyses further strengthened the robustness of our findings. The association between UMI and MACE was consistent across subgroups defined by age, sex, obesity grade, diabetes status, symptom status, LVEF, and the presence of myocardial ischemia. The absence of significant interactions suggests that the prognostic impact of UMI is broadly applicable across clinically relevant patient subsets, including those with preserved LVEF or minimal symptoms.

CMR offers unique advantages for the evaluation of cardiac function and detection of UMI, particularly in obese patients, in whom conventional diagnostic modalities may have reduced sensitivity and are often limited by body habitus [1,2,16,17,21]. As highlighted by Kramer, imaging-based approaches are essential for identifying MI that may be clinically silent or missed by conventional methods [6]. In addition, population-based CMR studies have demonstrated that UMI is common and carries adverse prognostic implications comparable to clinically recognized MI, underscoring the importance of accurate imaging-based detection [2]. In contrast, LGE CMR provides high spatial resolution and superior tissue characterization, allowing reliable identification of myocardial scar even in asymptomatic individuals and enabling detection of small MIs that may be missed by conventional diagnostic modalities [6,21,22].

From a clinical perspective, our findings highlight the importance of detecting unrecognized myocardial injury in patients with obesity. The presence of UMI may identify a subgroup of patients at particularly high risk for adverse outcomes who may benefit from aggressive risk factor modification and optimization of guideline-directed medical therapy. Nevertheless, further prospective studies are needed to determine whether management strategies guided by CMR-detected UMI can improve clinical outcomes. The cost-effectiveness and clinical utility of incorporating UMI assessment into routine care pathways remain to be established, particularly in resource-limited settings.

Regarding other imaging modalities, recent data also support the emerging role of computed tomography (CT) perfusion in patients with known or suspected CAD. A recent study by D’Ascenzo et al. showed that adding perfusion assessment to coronary CT angiography provides comparable clinical outcomes while improving selection for invasive evaluation and revascularization [23]. These findings highlight the potential value of integrating functional and anatomical imaging, although its role relative to CMR requires further investigation.

Several limitations should be acknowledged. First, this was a retrospective observational study conducted at a single tertiary referral center, which may limit generalizability. Second, all patients were referred for CMR, which may introduce referral bias and potentially overestimate the prevalence of UMI compared with the general population with obesity; however, ECG is not an ideal screening tool for prior MI, and this referral pattern likely reflects real-world clinical practice. Third, residual confounding cannot be entirely excluded despite multivariable adjustment, particularly with respect to ischemia burden and downstream revascularization. Patients with myocardial ischemia detected by CMR were more likely to undergo coronary revascularization, which could have influenced prognosis; however, analyses accounting for this factor did not materially affect the primary outcome. Finally, the relatively low number of events, especially cardiovascular death, may limit model stability and raise the possibility of overfitting.

In conclusion, in Thai patients with obesity undergoing CMR, the prevalence of UMI was 9.9% and was independently associated with long-term adverse cardiovascular outcomes, providing incremental prognostic information beyond traditional risk factors, with consistent effects across clinically relevant subgroups. These findings highlight the prognostic relevance of CMR-detected myocardial scar in this population and support further prospective, multicenter studies to clarify its role in risk assessment and clinical management.

## Supporting information

S1 Table Baseline characteristics of patients with normal BMI with and without UMI. (PDF)

S1 Fig Kaplan–Meier curves for MACE in patients with normal BMI. Kaplan–Meier curves showing survival free of MACE in patients with normal BMI, stratified by the presence or absence of UMI detected by CMR. Patients with UMI had a significantly higher incidence of MACE than those without UMI (log-rank p < 0.001). **Abbreviations:** BMI, body mass index; CMR, cardiac magnetic resonance; MACE, major adverse cardiovascular events; UMI, unrecognized myocardial infarction.
(PDF)

## Abbreviations

BMI: body mass index
CABG: coronary artery bypass graft;
CI: confidence interval
CMR: cardiac magnetic resonance
DBP: diastolic blood pressure
HR: hazard ratio
LGE: late gadolinium enhancement
LV: left ventricular
LVEDVI: left ventricular end-diastolic volume index
LVEF: left ventricular ejection fraction
LVESVI: left ventricular end-systolic volume index
MACE: major adverse cardiovascular events
ROC: receiver operating characteristic
SBP: systolic blood pressure
SSFP: steady-state free precession
TE: echo time
TR: repetition time
UMI: unrecognized myocardial infarction

## References

[1] Nordenskjöld AM, Hammar P, Ahlström H, Bjerner T, Duvernoy O, Lindahl B. Unrecognized myocardial infarction assessed by cardiac magnetic resonance imaging is associated with adverse long-term prognosis. PLoS One. 2018;13(7):e0200381. doi: 10.1371/journal.pone.0200381 29979788 PMC6034881

[2] Schelbert EB, Cao JJ, Sigurdsson S, Aspelund T, Kellman P, Aletras AH, et al. Prevalence and prognosis of unrecognized myocardial infarction determined by cardiac magnetic resonance in older adults. JAMA. 2012;308(9):890–6. doi: 10.1001/2012.jama.11089 22948699 PMC4137910

[3] Elliott MD, Heitner JF, Kim H, Wu E, Parker MA, Lee DC, et al. Prevalence and prognosis of unrecognized myocardial infarction in asymptomatic patients with diabetes: a two-center study with up to 5 years of follow-up. Diabetes Care. 2019;42(7):1290–6. doi: 10.2337/dc18-2266 31010876 PMC6973647

[4] Nogami K, Hoshino M, Kanaji Y, Sugiyama T, Misawa T, Hada M, et al. Prognostic implications of unrecognized myocardial infarction before elective percutaneous coronary intervention. Sci Rep. 2022;12(1):21579. doi: 10.1038/s41598-022-26088-z 36517567 PMC9751065

[5] Arai AE, Schulz-Menger J, Shah DJ, Han Y, Bandettini WP, Abraham A, et al. Stress perfusion cardiac magnetic resonance vs SPECT imaging for detection of coronary artery disease. J Am Coll Cardiol. 2023;82(19):1828–38. doi: 10.1016/j.jacc.2023.08.046 37914512

[6] Kramer CM. Detecting unrecognized myocardial infarction: the importance of imaging. Curr Cardiol Rep. 2010;12(1):3–5. doi: 10.1007/s11886-009-0079-8 20425177 PMC2999355

[7] Jensen CJ, Hayes B, Parker M, Wagner A, Lombardi M, Schwitter J. Relationship between obesity and unrecognized myocardial infarction: a EuroCMR multi-center study. J Cardiovasc Magn Reson. 2013;15(Suppl 1):O76. doi: 10.1186/1532-429X-15-S1-O76

[8] World Health Organisation. The Asia-Pacific perspective: redefining obesity and its treatment. 2000.

[9] Haam J-H, Kim BT, Kim EM, Kwon H, Kang J-H, Park JH, et al. Diagnosis of obesity: 2022 update of clinical practice guidelines for obesity by the Korean Society for the Study of Obesity. J Obes Metab Syndr. 2023;32(2):121–9. doi: 10.7570/jomes23031 37386771 PMC10327686

[10] Hicks KA, Mahaffey KW, Mehran R, Nissen SE, Wiviott SD, Dunn B, et al. 2017 Cardiovascular and Stroke endpoint definitions for clinical trials. Circulation. 2018;137(9):961–72. doi: 10.1161/CIRCULATIONAHA.117.033502 29483172

[11] Kaolawanich Y, Boonyasirinant T. Prognostic value of adenosine stress perfusion cardiac magnetic resonance imaging in older adults with known or suspected coronary artery disease. Arq Bras Cardiol. 2022;119(1):97–106. doi: 10.36660/abc.20210530 35830106 PMC9352122

[12] Kramer CM, Barkhausen J, Bucciarelli-Ducci C, Flamm SD, Kim RJ, Nagel E. Standardized cardiovascular magnetic resonance imaging (CMR) protocols: 2020 update. J Cardiovasc Magn Reson. 2020;22(1):17. doi: 10.1186/s12968-020-00607-1 32089132 PMC7038611

[13] Schulz-Menger J, Bluemke DA, Bremerich J, Flamm SD, Fogel MA, Friedrich MG, et al. Standardized image interpretation and post-processing in cardiovascular magnetic resonance - 2020 update. Journal of Cardiovascular Magnetic Resonance. 2020;22(1):19. doi: 10.1186/s12968-020-00610-6 32160925 PMC7066763

[14] Cerqueira MD, Weissman NJ, Dilsizian V, Jacobs AK, Kaul S, Laskey WK, et al. Standardized myocardial segmentation and nomenclature for tomographic imaging of the heart. A statement for healthcare professionals from the Cardiac Imaging Committee of the Council on Clinical Cardiology of the American Heart Association. Circulation. 2002;105(4):539–42. doi: 10.1161/hc0402.102975 11815441

[15] Niedziela J, Hudzik B, Niedziela N, Gąsior M, Gierlotka M, Wasilewski J, et al. The obesity paradox in acute coronary syndrome: a meta-analysis. Eur J Epidemiol. 2014;29(11):801–12. doi: 10.1007/s10654-014-9961-9 25354991 PMC4220102

[16] Kwong RY, Chan AK, Brown KA, Chan CW, Reynolds HG, Tsang S, et al. Impact of unrecognized myocardial scar detected by cardiac magnetic resonance imaging on event-free survival in patients presenting with signs or symptoms of coronary artery disease. Circulation. 2006;113(23):2733–43. doi: 10.1161/CIRCULATIONAHA.105.570648 16754804

[17] Antiochos P, Ge Y, Steel K, Bingham S, Abdullah S, Mikolich JR, et al. Imaging of clinically unrecognized myocardial fibrosis in patients with suspected coronary artery disease. J Am Coll Cardiol. 2020;76(8):945–57. doi: 10.1016/j.jacc.2020.06.063 32819469 PMC8691844

[18] Ebong IA, Goff Jr DC, Rodriguez CJ, Chen H, Bertoni AG. Mechanisms of heart failure in obesity. Obes Res Clin Pract. 2014;8(6):e540-8. doi: 10.1016/j.orcp.2013.12.005 25434909 PMC4250935

[19] Chandramouli C, Tay WT, Bamadhaj NS, Tromp J, Teng T-HK, Yap JJL, et al. Association of obesity with heart failure outcomes in 11 Asian regions: A cohort study. PLoS Med. 2019;16(9):e1002916. doi: 10.1371/journal.pmed.1002916 31550265 PMC6759142

[20] Sakboonyarat B, Poovieng J, Rangsin R. Association between obesity and new-onset heart failure among patients with hypertension in Thailand. J Health Popul Nutr. 2024;43(1):33. doi: 10.1186/s41043-024-00530-6 38424593 PMC10905941

[21] Andrade JM, Gowdak LHW, Giorgi MCP, de Paula FJ, Kalil-Filho R, de Lima JJG, et al. Cardiac MRI for detection of unrecognized myocardial infarction in patients with end-stage renal disease: comparison with ECG and scintigraphy. AJR Am J Roentgenol. 2009;193(1):W25-32. doi: 10.2214/AJR.08.1389 19542379

[22] Arai AE. Magnetic resonance imaging for area at risk, myocardial infarction, and myocardial salvage. J Cardiovasc Pharmacol Ther. 2011;16(3–4):313–20. doi: 10.1177/1074248411412378 21821534 PMC8690274

[23] D’Ascenzo F, Faletti R, Di Pietro G, Improta R, Bruno F, Schoepf UJ, et al. Coronary CT angiography alone versus with CT perfusion: a systematic review and meta-analysis assessing approaches for chest pain. Eur Radiol. 2025;35(9):5501–13. doi: 10.1007/s00330-025-11459-7 40055231

