# Supplementary material for: Prevalence and prognostic impact of unrecognized myocardial infarction detected by cardiac magnetic resonance in Thai patients with obesity
Source: PLoS One. 2026 Jul 6;21(7):e0353109. doi: 10.1371/journal.pone.0353109 (PMC13336170; doi:10.1371/journal.pone.0353109)
Supplement: S1 Table — (PDF) [file pone.0353109.s001.pdf]

S1 Table Baseline characteristics of patients with normal BMI with and without UMI.

	Total (n=191)	UMI (n=31)	No UMI (n=160)	P-value
Age, years	66.1±13.3	66.0±11.0	66.1±13.7	0.96
Male	102 (53.4)	27 (87.1)	75 (46.9)	***<0.001***
BMI, kg/m^2	22.3±2.1	22.4±1.9	22.3±2.1	0.91
Hypertension	132 (69.1)	20 (64.5)	112 (70.0)	0.54
Diabetes mellitus	56 (29.3)	12 (38.7)	44 (27.5)	0.21
Hyperlipidemia	116 (60.7)	19 (61.3)	97 (60.6)	0.94
Family history of CAD	3 (1.6)	0 (0.0)	3 (1.9)	>0.99
Cigarette smoking	17 (8.9)	6 (19.4)	11 (6.9)	***0.03***
History of heart failure	27 (14.1)	7 (22.6)	20 (12.5)	0.16
Atrial fibrillation	18 (9.4)	2 (6.5)	16 (10.0)	0.74
Ischemic stroke	14 (7.3)	4 (12.9)	10 (6.2)	0.25
Chest pain	70 (36.6)	12 (38.7)	58 (36.2)	0.79
Dyspnea	123 (64.4)	18 (58.1)	105 (65.6)	0.42
Medications				
Aspirin	80 (41.9)	16 (51.6)	64 (40.0)	0.23
ACE inhibitor or ARB	64 (33.5)	13 (41.9)	51 (31.9)	0.27
Beta blocker	75 (39.3)	13 (41.9)	62 (38.8)	0.74
Calcium channel blocker	47 (24.6)	4 (12.9)	43 (26.9)	0.09
Statin	78 (40.8)	14 (45.2)	64 (40.0)	0.59
Oral antidiabetic drug	27 (14.1)	6 (19.4)	21 (13.1)	0.39
Insulin	3 (1.6)	1 (3.2)	2 (1.2)	0.41
CMR				
LVEDV index, mL/m^2	85.1±39.4	113.4±64.6	79.6±29.6	***0.007***
LVESV index, mL/m^2	23.1 (17.0,33.8)	46.1 (24.1,81.0)	21.8 (16.5,29.3)	***<0.001***
LVEF, %	64.2±15.9	52.6±19.3	66.5±14.1	***0.001***
Presence of myocardial ischemia	34 (17.8)	19 (61.3)	15 (9.4)	***<0.001***
Number of segments of myocardial ischemia [a]	4.5 (3,6)	4 (3,6)	5 (2,7)	>0.99
Number of segments of UMI [b]	4 (2,7)	4 (2,7)	0 (0,0)	***<0.001***

Data are presented as mean±standard deviation, median (interquartile range), or number (percentage), as appropriate.
Bold italic values indicate statistical significance (p<0.05).
[a] Data available in patients with myocardial ischemia.
[b] Data available in patients with UMI.

Abbreviations: ACE, angiotensin-converting enzyme; ARB, angiotensin receptor blocker; BMI, body mass index; CAD, coronary artery disease; CMR, cardiac magnetic resonance; LVEDV, left ventricular end-diastolic volume; LVESV, left ventricular end-systolic volume; LVEF, left ventricular ejection fraction; UMI, unrecognized myocardial infarction.
