# Supplementary material for: Prevalence and prognostic impact of unrecognized myocardial infarction detected by cardiac magnetic resonance in Thai patients with obesity
Source: PLoS One. 2026 Jul 6;21(7):e0353109. doi: 10.1371/journal.pone.0353109 (PMC13336170; doi:10.1371/journal.pone.0353109)
Supplement: S1 Fig — Kaplan–Meier curves showing survival free of MACE in patients with normal BMI, stratified by the presence or absence of UMI detected by CMR. Patients with UMI had a significantly higher incidence of MACE than those without UMI (log-rank p < 0.001). Abbreviations: BMI, body mass index; CMR, cardiac magnetic resonance; MACE, major adverse cardiovascular events; UMI, unrecognized myocardial infarction. (PDF) [file pone.0353109.s002.pdf]

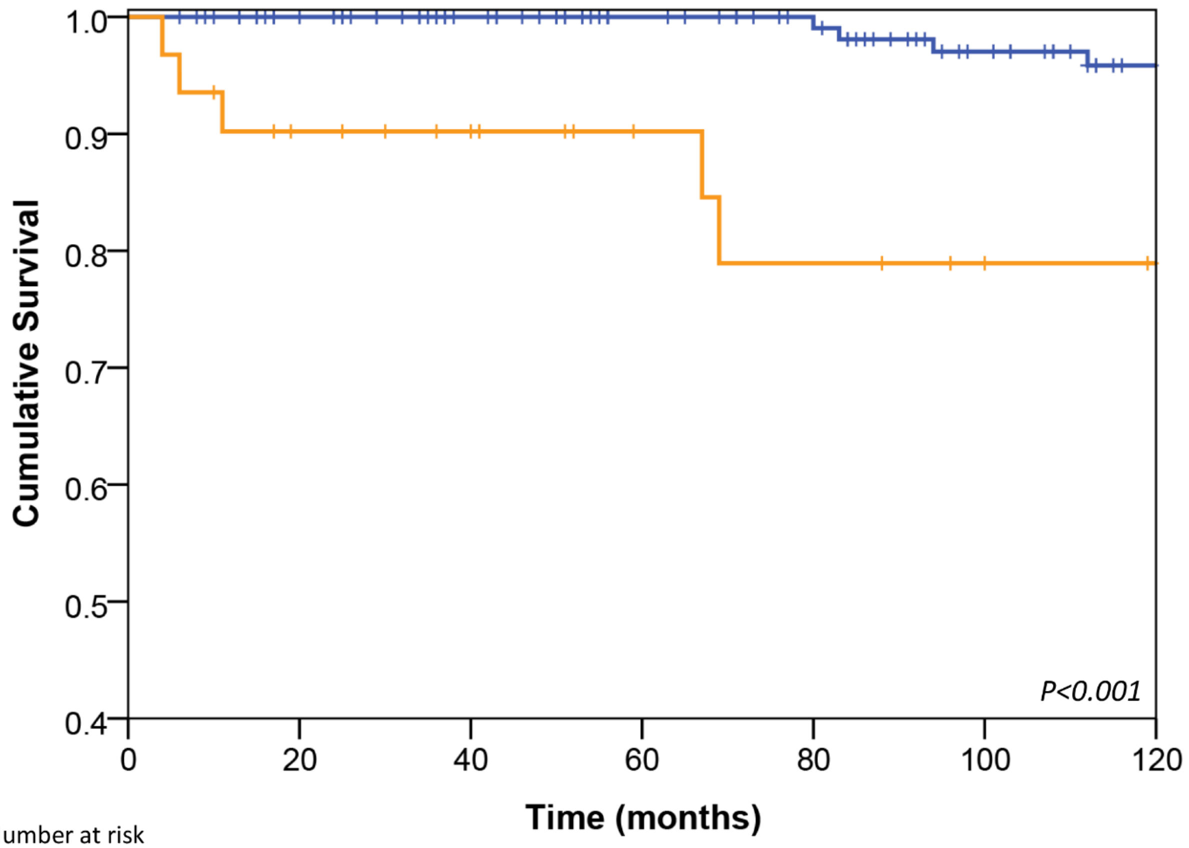

Number at risk

No UMI	160	147	130	113	105	89	76
UMI	31	25	22	16	14	12	10

S1 Figure Kaplan–Meier curves for MACE in patients with normal BMI.

Kaplan–Meier curves showing survival free of MACE in patients with normal BMI, stratified by the presence or absence of UMI detected by CMR. Patients with UMI had a significantly higher incidence of MACE than those without UMI (log-rank $p<0.001$).

Abbreviations: BMI, body mass index; CMR, cardiac magnetic resonance; MACE, major adverse cardiovascular events; UMI, unrecognized myocardial infarction.
